# Outcomes of Subpectoral Tenodesis in the Treatment of Proximal Biceps Pathologies

**DOI:** 10.7759/cureus.76360

**Published:** 2024-12-25

**Authors:** Bernardo P Hespanhol, Armando Secundino, Filipe Baracho, Carina Cohen, Fernanda Piva, Pedro Machado

**Affiliations:** 1 Shoulder Surgery, Hospital do Trabalhador, Curitiba, BRA

**Keywords:** biceps tenodesis, long head of biceps tendon, proximal biceps, slap lesions, subpectoral tenodesis

## Abstract

Introduction

The aim of this article is to evaluate the clinical and functional outcomes of subpectoral tenodesis of the long head of the biceps (LHB) in the treatment of proximal biceps pathologies.

Methods

A retrospective, cross-sectional study was conducted through the analysis of medical records from 24 patients and 26 shoulders who underwent the subpectoral tenodesis technique using bone tunnels. Three patients were excluded due to insufficient data to calculate the functional scores. The final study sample comprised 21 patients and 23 shoulders. Clinical and functional outcomes were evaluated based on the American Shoulder and Elbow Surgeons (ASES) Score and the Long Head of Biceps (LHB) Score. Additionally, the main indications for the procedure and potential complications were thoroughly assessed.

Results

The average follow-up time was four years and, of the 21 patients (23 shoulders), 19 (82.6%) were male and 4 (17.4%) were female shoulders, with a mean age of 41.6 years. The main indication for surgery was superior labral anterior to posterior injury in 12 (52.2%) cases, followed by subluxation and tenosynovitis. No case presented aesthetic deformity or pain on palpation at the LHB. Patients showed significant improvement in the ASES score (average from 75.13 to 98.52) and LHB score (average from 68.82 to 98.34).

Conclusion

The subpectoral tenodesis technique is a safe procedure, with good clinical and functional outcomes for the treatment of proximal biceps pathologies.

## Introduction

Pathologies related to the long head of the biceps (LHB) are frequent causes of anterior shoulder pain [1]. These include subluxation or instability, tears, superior labral anterior to posterior (SLAP) lesions, and tenosynovitis [2]. 

Additionally, these conditions are often associated with other diseases, which makes the diagnosis challenging [3]. Various maneuvers during the physical examination have been described to isolate pain related to LHB pathology [4], although many studies question their accuracy. Thus, diagnostic elucidation often requires complementary imaging tests [5].

Conservative treatment is typically attempted initially [6]. However, if this approach fails, surgical treatment should be proposed, which consists of two options: tenotomy or tenodesis. There still remains considerable controversy in the literature regarding the best option, as some studies show no clinical difference between the two techniques [7], while others highlight persistent local pain, cramping, loss of strength, aesthetic deformity, and patient dissatisfaction following tenotomy [8]. 

In this context, tenodesis has become the preferred option, especially for young and high-demand patients. It can be performed using various fixation techniques [9] (anchors, interference screws, bone tunnels, or soft tissue sutures) [10-14] and at different locations (glenoid, lesser tuberosity, bicipital groove, near the pectoralis major, or subpectoral) [15-18]. 

There is no consensus on the ideal location for tenodesis. However, subpectoral tenodesis offers notable advantages, such as restoring the anatomical length-tension relationship of the biceps muscle by anchoring it to the inferior edge of the pectoralis major tendon and completely removing the diseased portion of the LHB from the bicipital groove, thereby reducing the risk of residual local pain. The method selected by the authors of this study for treating LHB pathologies was subpectoral tenodesis, performed using bone tunnels, LHB imbrication, and fixation sutures.

The primary aim of this study is to evaluate the clinical and functional outcomes of this technique, as well as complications and main indications for its use. The secondary aim is to describe the procedure in detail, emphasizing its practicality, ease of application, and potential for widespread use in achieving favorable clinical outcomes. The study hypothesizes that this approach replicates the positive results observed in other studies using implant fixation techniques, with improved functional scores and low complication rates [11,13,14,16].

## Materials and methods

This retrospective, cross-sectional study was conducted through the analysis of medical records from 24 patients and 26 shoulders. The inclusion criteria were as follows: patients who underwent shoulder arthroscopy and subpectoral tenodesis using bone tunnels, both performed in the same surgical procedure by the responsible surgical team between April 2015 and June 2019. Additional criteria included patients aged over 18 years and with a minimum follow-up duration of two years. The exclusion criteria were those lacking complete pre- and post-operative medical records; individuals under 18 years; patients with a follow-up period shorter than two years; those with a full-thickness rotator cuff tear; or patients lost to follow-up.

Three patients were excluded due to insufficient data to calculate the functional scores. The final study sample comprised 21 patients and 23 shoulders. The indications for tenodesis included SLAP type III and IV lesions, tenosynovitis, and subluxation of the long head of the biceps.

The patients were evaluated according to the American Shoulder and Elbow Surgeons (ASES) score and the Long Head of Biceps (LHB) score, with pain and dynamometer measurements incorporated into the scoring system. The strength of flexion was measured with a handheld digital dynamometer (microFET®2 Digital Handheld Dynamometer, Hoogan Scientific, Salt Lake City, USA), with the elbow at 90 degrees of flexion, in both supinated and neutral positions, allowing for comparison between the upper limbs. Additionally, the presence of aesthetic deformity and residual pain along the path of the LHB tendon on palpation of the bicipital groove was also evaluated.

Furthermore, patients were analyzed based on the side of the surgery, sex, physical activity level, and follow-up duration.

The comparison between two groups regarding quantitative variables was conducted using the Mann-Whitney nonparametric test, with the groups being the pre-operative and post-operative scores for both the ASES and LHB. This test was used when the data did not exhibit a normal distribution. The level of statistical significance was set at 5%, and the data were analyzed using R version 4.2.2 statistical analysis software (R Foundation for Statistical Computing, Vienna, Austria).

This study was approved by the ethics committee of the institution with the approval number 5.530.804.

Surgical technique

The patients were positioned in a lateral decubitus position with the limb in longitudinal traction. Initially, the LHB was repaired using one high-strength Hi-Fi® Suture (CONMED Corporation, Largo, USA) and tenotomy was performed at its insertion. After the traction was released, a small incision was made at the axillary fold. Dissection was carried out to identify and retrieve the LHB along with the anterior portion of the humerus. A modified Kracow suture was performed, starting 1 cm distal to the myotendinous junction and extending 3 cm distally, with excess tendon being resected. 

**Figure 1:**
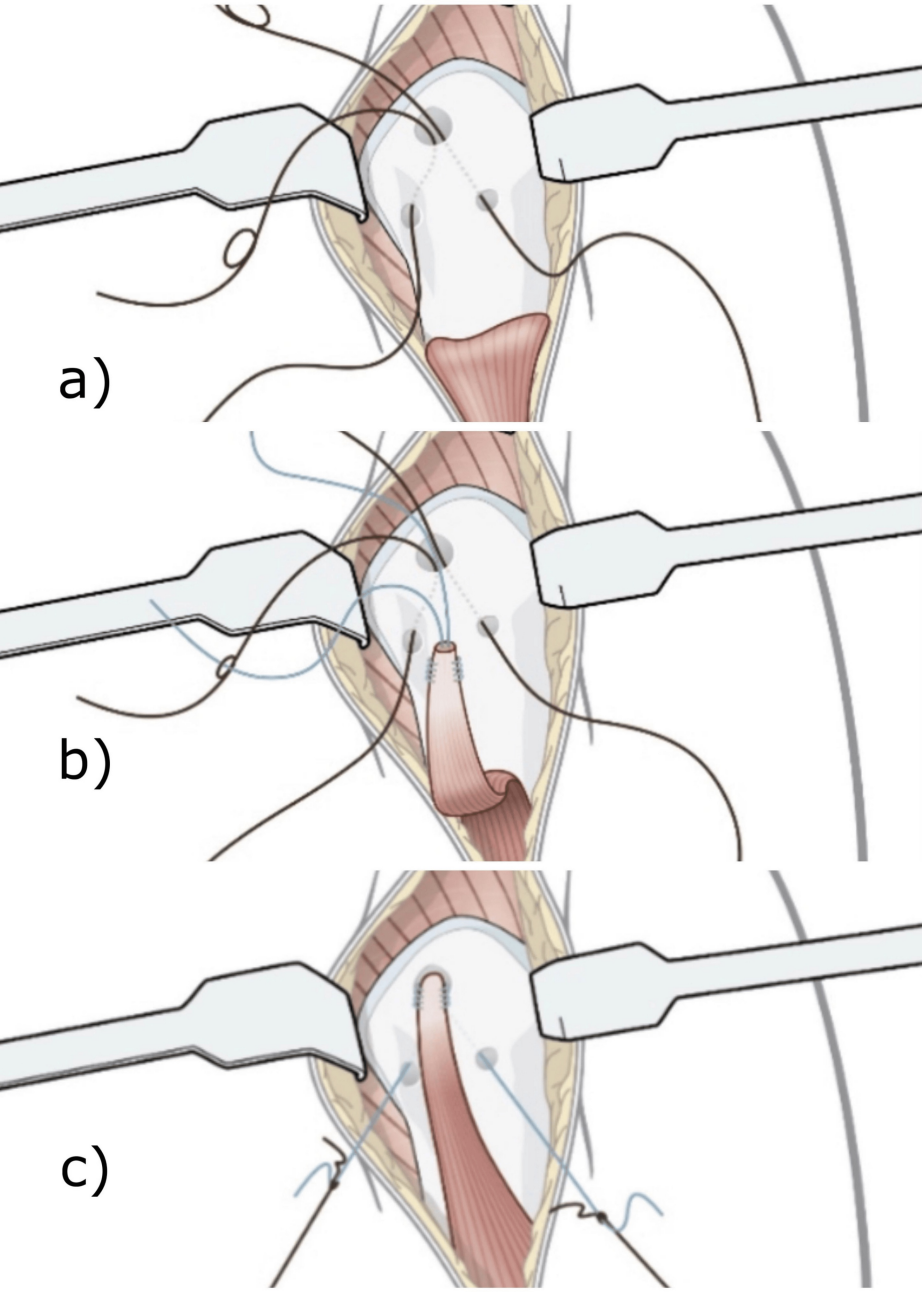
Step-by-step guide to the bone tunnel suture passage a) Transport sutures; b) Transportation of the high-strength suture through the holes; c) Introduction of the long head of biceps into the central hole after tensioning the suture Image credit: Authors

Next, a unicortical superior central hole was created just above the inferior border of the pectoralis major using a 5.5mm or 6.0mm drill. Then, two lower unicortical perforations were made inferior to the central hole, forming a triangle with a 1 cm distance between the holes, using a Kirschner wire or a 2.5mm drill. High-strength sutures from the LHB were passed through the central hole and retrieved via the two inferior holes using a needle and non-absorbable suture for transport (Figure 1a).

The LHB tendon was introduced into the central hole, and with the elbow at 90° of flexion, appropriate tensioning, and suturing were performed (Figure 1b and Figure 1c). 

In the postoperative period, the patient wore a sling for four weeks, with passive and active elbow mobility allowed and pendulum shoulder movements permitted. After four weeks, exercises to gain shoulder mobility and isometric elbow flexion exercises were started, and eccentric load activities were permitted after eight weeks, gradually. 

## Results

A total of 23 shoulders in 21 patients who underwent tenodesis were included in the study. The average follow-up time was four years. Among the 23 shoulders, 19 (82.6%) were male patients and 4 (17.4%) were female patients, with a mean age of 41.6 years. 

The main indication for surgery was SLAP lesion in 12 (52.2%) cases, followed by subluxation in eight (34.8%) cases and tenosynovitis in three (13%) cases (Table 1). 

**Table 1:** Sample Description The data has been represented as N and %. Mean±SD, minimum values and maximum values (min-max) for age. SD: Standard deviation; SLAP: superior labral anterior to posterior; LHB: long head of the biceps

Variables			Total (n= 23)
				Male - n(%)			19 (82.6)
Mean age ± SD; (Min – Max)			41.57 ± 11.9 ; (26 – 62)
Age– n(%)	20 – 29		2(8.7)
	30 – 39		10(43.5)
	40 – 49		5(21.7)
	50 years or more		6(26.1)
Right handed– n(%)			19(82.6)
Side of surgery (right) – n(%)			16(69.6)
Physical activity – n(%)		Only one	15 (65.2)
		Two or more	8 (34.8)
Follow up – n(%)		Two years	6(26.1)
		Three years	9(39.1)
		Four years or more	8(34.8)
Injury - n(%)	SLAP III		9(39.1)
	SLAP IV		3(13)
	LHB subluxation		8(34.8)
	Tenosynovitis		3(13)

In the clinical evaluation, the aesthetic deformity was classified as absent, mild, moderate, or severe, as suggested by Scheibel et al. [16]. All patients presented no deformity. No cases showed pain along the path of the long head of the biceps on palpation of the bicipital groove. The comparison of the average functional scores (ASES and LHB) pre- and postoperatively showed significant improvement, as shown in Tables 2, 3. The ASES score increased from 75.13 to 98.52 (Table 2), and the LHB score improved from 68.82 to 98.34 (Table 3), both with statistical significance (p<0.001). 

**Table 2:** ASES Score Comparison The data has been presented with mean +- standard deviation (SD), minimum values and maximum values (min-max) The Wilcoxon Mann-Whitney nonparametric test was used to find the p-value ASES: American Shoulder and Elbow Surgeons

ASES Score	Measure	p-value	Statistic Test
Preoperative- Mean± SD (Min – Max)	75.1 ± 5.8 (67 – 82)	<0.001	Wilcoxon Mann-Whitney
Postoperative- Mean ± SD (Min – Max)	98.5 ± 1.8 (92 – 100)		Wilcoxon Mann-Whitney

**Table 3:** LHB Score Comparison The data has been presented as mean +- standard deviation (SD), minimum values and maximum values (min-max) The Wilcoxon Mann-Whitney nonparametric test was used to find the p-value LHB: Long head of the biceps

LHB Score	Measure	p-value	Statistic Test
Preoperative Mean ± SD (Min – Max)	68.8 ± 3.6 (64 – 73)	<0.001	Wilcoxon Mann-Whitney
Postoperative Mean ± SD (Min – Max)	98.3 ± 1.7 (94 – 100)		Wilcoxon Mann-Whitney

## Discussion

There are currently no Level I or II studies comparing the ideal location for tenodesis, whether above, within, or below the bicipital groove. However, subpectoral tenodesis has the advantage of removing the LHB tendon from its groove, a site often affected in proximal biceps pathologies, thus eliminating potential residual pain associated with stenosis or tenosynovitis. One of the objectives of this article is to demonstrate that the subpectoral tenodesis technique, performed using bone tunnels, improves both clinical and functional scores, in part by reducing pain. This finding aligns with the results reported by Lutton et al., who performed suprapectoral arthroscopic tenodesis along the bicipital groove and suggested that more distal fixation of the LHB tendon is associated with lower rates of postoperative pain [18].

Subpectoral LHB tenodesis is a safe, reliable, and effective treatment for proximal biceps pathologies [13]. It provides symptom relief, functional improvement, and low complication rates. Millet et al. followed 88 patients who underwent subpectoral LHB tenodesis with interference screws or anchors for an average of 13 months, reporting improvements in functional scores (ASES from 28 to 76, and Constant Modified from 29 to 59) with no failures [11]. The present study also used a technique similar to that of Millet et al., performing subpectoral LHB tenodesis but without the use of implants such as screws or anchors. Despite this difference, the study reproduced the functional improvements observed in the study by Millet et al., further supporting the effectiveness of the subpectoral tenodesis technique.

There is still considerable controversy regarding the best management of SLAP type II lesions in young and active patients. Boileau et al. reported excellent results, with 87% of patients with SLAP type II lesions returning to their pre-injury sports level after arthroscopic LHB tenodesis with interference screw [12]. In a prospective study of 101 active military patients (40 with SLAP type II lesions and 61 with LHB tenosynovitis), Provencher et al. demonstrated good results after subpectoral tenodesis with interference screw, showing a significant increase in Western Ontario rotator cuff (WORC) scores from 54% to 89% and Single Assessment Numeric Evaluation (SANE) from 58 to 89.5 (p < 0.01). Nearly all patients returned to their desired activity levels, with 82% returning to work and physical activities about four months after the procedure [15].

Although SLAP type II lesions were not an indication in the present study, previous research suggests that they can yield good results when treated with other techniques. As demonstrated by Boileau et al. and Provencher et al., LHB tenodesis can be effective for these lesions [12,15]. The present study suggests that subpectoral tenodesis using bone tunnels yields good clinical and functional outcomes for classical indications. For a select group of patients, such as overhead athletes over 35 years old or those who may benefit from LHB tenodesis due to work-related activities, this technique could also be a viable option.

The main findings of this study demonstrate that subpectoral tenodesis of the LHB using bone tunnels is a minimally invasive and highly effective technique, with significant improvement in the ASES and LHB functional scores. 

Although this study corroborated the positive results of other studies on subpectoral tenodesis, we identified some limitations. The sample size was small, and the average follow-up period was only four years. Additionally, imaging tests such as ultrasound and MRI were not performed to assess the integrity of LHB fixation; only clinical evaluation was conducted. Finally, larger studies with longer follow-up periods are needed to define the ideal location for LHB tenodesis, the most effective fixation technique, and whether favorable results are maintained over time. 

## Conclusions

The subpectoral biceps tendon tenodesis technique, performed using bone tunnels, has been demonstrated to be a safe and effective procedure, offering significant clinical and functional improvements in patients treated for various proximal biceps pathologies, including SLAP lesions, subluxation, and tenosynovitis. The findings revealed substantial enhancements in ASES and LHB scores, indicating both functional recovery and patient satisfaction. Furthermore, there were no reports of aesthetic deformity or residual pain along the path of the biceps tendon, supporting the effectiveness of the procedure in achieving symptom relief and functional recovery.
